# Prevalence and predictive factors of osteoporosis in systemic sclerosis patients: a case-control study

**DOI:** 10.18632/oncotarget.3806

**Published:** 2015-04-12

**Authors:** Mathilde Marot, Antoine Valéry, Eric Esteve, Guido Bens, Adelheid Müller, Stéphanie Rist, Hechmi Toumi, Eric Lespessailles

**Affiliations:** ^1^ Service de Rhumatologie, Centre Hospitalier Universitaire de Tours, France; ^2^ Service du D.I.M., Centre Hospitalier Régional d'Orléans, La Source, France; ^3^ Service de Dermatologie, Centre Hospitalier Régional d'Orléans, Porte Madeleine; ^4^ Univ. Orléans, Orleans, France; ^5^ Service de Rhumatologie, Centre Hospitalier Régional d'Orléans, La Source, France

**Keywords:** osteopenia, densitometry, trabecular bone, chronic inflammatory, rheumatic diseases

## Abstract

**Purpose:**

Investigate the prevalence of osteoporosis in patients with systemic sclerosis (SSc) and describe alterations of bone tissue with High-Resolution peripheral Quantitative Computed Tomography (HR-pQCT).

**Methods:**

Thirty-three patients and 33 controls matched on age, body mass index (BMI) and menopause were included. Bone mineral density (BMD) was measured at the lumbar spine (LS), femoral neck (FN) and total hip (TH) by dual energy X-ray absorptiometry. Volumetric BMD (vBMD) and bone microarchitecture were measured by HR-pQCT at tibia and radius.

**Results:**

In patients, BMI was significantly lower, the prevalence of osteoporosis was significantly higher and HR-pQCT analysis showed a significant alteration of the trabecular compartment with a decrease in trabecular vBMD on both sites than in controls. In multivariate analysis, a low lean body mass, presence of anticentromere antibodies and older age were identified as independent factors for decreased BMD at LS (r²=0.43), FN (r²=0.61) and TH (r²=0.73). History or current digital ulcers were also identified as an independent factor for microarchitecture alteration.

**Conclusion:**

In patients an increased prevalence of osteoporosis was found and HR-pQCT showed impaired trabecular bone compartment. Also, low lean body mass, high age, digital ulcers and ACAs were identified as independent risk factors for bone damage.

## INTRODUCTION

Systemic sclerosis (SSc) is a chronic connective tissue disease characterized both by fibrosis and endothelial dysfunction [1].

Although the extent of skin involvement is the key feature of this common disease, joint, periarticular soft tissue and bone may be involved, leading to severe alteration of the quality of life [2] in particular when hands and feet are affected by the disease [3]. Although articular manifestations are frequent and well described in SSc [4], the suggestion that SSc may be associated with bone loss and osteoporosis (OP) is still debated.

Chronic inflammatory rheumatic diseases like rheumatoid arthritis and spondyloarthropathy are well recognized risk factors of bone loss and fractures [5]. SSc has been recognized to be another potential inflammatory joint diseases that may affect bone tissue [6-8]. Currently, there are no recommendations for the systematic search of OP in SSc [9]. OP is typically diagnosed by dual-energy x-ray absorptiometry (DXA) measurements of areal Bone Mineral Density (BMD). However, OP is characterized by low bone mass but also by bone quality alterations both leading to aincreased risk of fracture. High-Resolution peripheral Quantitative Computed Tomography (HR-pQCT) is a non-invasive imaging technique that can measure volumetric BMD (vBMD) and microarchitecture parameters of cortical and trabecular bone [10]. This tool has been used in clinical studies to investigate differences in bone microarchitecture between patients with or without fractures [11, 12]. It was also recently used in patients with systemic lupus erythematosus to investigate the microarchitecture deterioration associated with long-term glucorticoid treatment [13]. The aim of our study was to investigate the prevalence of OP by conventional DXA, in caucasian female patients with SSc compared to healthy controls. We also described the existence of qualitative disorders of bone tissue in SSc by HR-pQCT and further investigated the associations of some illness's characteristics with bone parameters.

## RESULTS

Statistical tests and calculations were performed on the thirty-three patients and 33 controls participants. As shown in Table 1, no significant difference was observed between patients and controls for all calculated parameters except for the BMI, which was significantly lower in patients compared to controls (*p* = 0.029). LS, TH and FN BMD, were significantly lower in patients with SSc compared with the control group (Table 2). After adjustment for BMI, a significant difference in BMD persisted at TH BMD. Among postmenopausal women (*n* = 28 in each group), the prevalence of OP in SSc patients (42.8 %) was significantly different than in controls (10.7%), (*p* < 0.05). Table 3 showed the prevalence of OP and osteopenia at each sites in postmenopausal subjects

**Table 1:** Characteristics of systemic sclerosis patients and their age, menopause and BMI matched paired controls *

Demographics			
Age, years (mean±SD)	63.2±10.2	62.9±10,4	NS
BMI, kg/m^2^ (mean±SD)	24.7±4.9	25.6±5.0	0.029
Duration of menopause, years (mean±SD)	15.4±11.4	13.9±9.7	NS
Menopause, n (%)	28 (85)	28 (85)	NS
Current smoking, n (%)	2 (6)	4 (12.1)	NS
Alcohol, n (%)	0 (0)	0 (0)	NS
Personal history of fracture, n (%)	10 (30.3)	5 (15.1)	NS
Family history of hip fracture, n (%)	5 (15.1)	3 (9.1)	NS
Treatment, n (%)			
Prednisone	6 (18.2)	0 (0)	
Osteoporosis treatment	12 (36.4)	0 (0)	
Calcium and / or vitamin D	20 (60.6)	18 (59.4)	
Inhibitor of proton pump	26 (78.8)	4 (12)	< 0.01
Methotrexate	2 (6)	0 (0)	
Hydroxychloroquine	7 (21.2)	0 (0)	
History of cyclic intravenous prostanoids	10 (30.3)	0 (0)	
Cyclophosphamide	0 (0)	0 (0)	
Biology, n (%)			
CRP (>10 mg/l)	1 (3)	ND	
Vitamin D deficiency (< 10 ng/ml)	2 (6)	ND	
Vitamin D insufficiency (10-30 ng/ml)	18 (54.5)	ND	
Parathyroid hormone (>61 ng/l)	4 (12.1)	ND	
Calcemia (<2.2 or >2.6 mmol/l)	0 (0)	ND	
ANA	33 (100)	ND	
ACA	20 (60.6)	ND	
Antitopoisomerase (anti-Scl70)	4 (12.1)	ND	
Anti-RNA polymerase III	0 (0)	ND	
Disease Characteristics			
Disease duration, years (mean±SD)	9.5±8.4	NA	
Skin involvment subset, n (%)			
limited cutaneous	26 (78.8)	NA	
diffuse cutaneous	3 (9.1)	NA	
limited sine scleroderma	4 (12.1)	NA	
Current digital ulcers, n (%)	12 (36.4)	NA	
History of digital ulcers, n (%)	21 (63.6)	NA	
Organ involvment, n (%)			
gastrointestinal involvment	24 (72.7)	NA	
malabsorption syndrome	0 (0)	NA	
lung disease	9 (27.2)	NA	
pulmonary hypertension	4 (12.1)	NA	
scleroderma renal crisis	0 (0)	NA	
joint damage	26 (78.8)	NA	
Raynaud's syndrome	33 (100)	NA	
HAQ (n = 32) (mean±SD)	0.833±0.830	NA	

* SD = standard deviation; n = number; HAQ = Health Assessment Questionnaire; BMI = body mass index; CRP = C reactive Protein; ANA = anti nuclear antibody; ACA = anticentromere antibody; ND = not determined; NA = not applicable.

**Table 2:** Results of bone mineral density (BMD) and body composition by dual energy x-ray absorptiometry (DXA) in women with systemic sclerosis and in controls*

	Patients (mean±SD)	Controls (mean±SD)	p	p (adjusted for BMI)
LS BMD (g/cm^2^)	0.918 ± 0.157 (n=32)	0.941 **±** 0.138 (n=33)	0.0164	0.176
FN BMD (g/cm^2^)	0.694 **±** 0.122 (n=33)	0.736 **±** 0.113 (n=33)	0.0123	0.129
TH BMD (g/cm^2^)	0.796 **±** 0.127 (n=33)	0.867 ± 0.105 (n=33)	0.0002	< 0.02
Lean mass (kg)	35.69 **±** 6.15 (n=30)	38.52 **±** 5.79 (n=29)	NS	
Fat mass (kg)	21.21 **±** 10.02 (n=30)	24.61 **±** 9.15 (n=29)	NS	

* The results are expressed as the mean±standard deviation (SD) of densitometric values in g/cm2 and values of lean and fat mass in kilograms (kg). NS = not significant; n = number of subjects; LS = lumbar spine; FN = femoral neck; TH = total hip.

**Table 3:** Results of the prevalence of osteoporosis and osteopenia in postmenopausal women with systemic sclerosis and controls, as classified by the World Health Organization

	Patients	Controls	p
Lumbar spine (%)			
Osteoporosis	33.3 (n = 27)	7.1 (n = 28)	< 0.05
Osteopenia	25.9 (n = 27)	46.4 (n = 28)	NS
Femoral neck (%)			
Osteoporosis	10.7 (n = 28)	3.6 (n = 28)	NS
Osteopenia	64.2 (n = 28)	64.2 (n = 28)	-
Total hip (%)			
Osteoporosis	7.1 (n = 28)	0 (n = 28)	NS
Osteopenia	50 (n = 28)	39.3 (n = 28)	NS

Among premenopausal women none had Z-score < - 2 DS

Body composition parameters were similar between the two groups (data not shown)

Evaluation by HR-pQCT showed that trabecular bone quality was significantly impaired in patients compared to controls (Table 4). In SSc patients, we found an alteration of trabecular bone compartment with a significant decrease in Dtrab (approximately −17%) and BV/TV (approximately −15%) at both sites. In addition, we found alterations of the trabecular microarchitecture with a significant decrease in Tb.Th at radius (−8%), increased Tb.Sp (+ 15%) and decreased Tb.N at tibia (−10.7%). However, measures related to the evaluation of the cortical compartment (DCort and Ct.Th) were comparable between the groups.

**Table 4:** Bone densitometric and microarchitectural variables by the HR-pQCT in women with systemic sclerosis and in controls*

Variables	Patients (n=33)	Controls (n=33)	p value (adjusted for BMI)
Distal radius			
Dcort (mg HA/cm^3^)	836.5 ± 86.7	826.3 ± 79.6	0.270
Dtrab (mg HA/cm^3^)	126.5 ± 41.9	153.8 ± 42.4	< 0.02
D100 (mg HA/cm^3^)	287.8 ± 75.8	300.7 ± 70.8	0.371
Dinn (mg HA/cm^3^)	86.7 ± 45.5	115.3 ± 43.3	< 0.02
Dmeta (mg HA/cm^3^)	183.9 ± 39.1	209.5 ± 43.0	< 0.02
Ct.Th (mm)	0.7 ± 0.22	0.70 ± 0.19	0.306
BV/TV	0.1 ± 0.04	0.13 ± 0.03	< 0.02
Tb.N (1/mm)	1.6 ± 0.38	1.72 ± 0.33	0.081
Tb.Th (mm)	0.067 ± 0.01	0.073 ± 0.01	< 0.02
Tb.Sp (mm)	0.62 ±0.23	0.53 ± 0.16	0.073
Tb1.NSD (mm)	0.32 ± 0.23	0.27 ± 0.17	0.243
Tibia			
Dcort (mg HA/cm^3^)	815.5 ± 71.8	802.8 ± 83.7	0.165
Dtrab (mg HA/cm^3^)	143.3 ± 44.4	170.8 ± 36.8	< 0.02
D100 (mg HA/cm^3^)	257.2 ± 62.7	275.5 ± 53.5	0.182
Dinn (mg HA/cm^3^)	98.3 ± 48.2	128.1 ± 39.9	< 0.02
Dmeta (mg HA/cm^3^)	209.5 ± 43.4	233.7 ± 34.4	< 0.02
Ct.Th (mm)	0.98 ± 0.26	0.97 ± 0.30	0.343
BV/TV	0.12 ± 0.04	0.14 ± 0.03	< 0.02
Tb.N (1/mm)	1.57 ± 0.33	1.76 ± 0.27	< 0.02
Tb.Th (mm)	0.08 ± 0.02	0.08 ± 0.01	0.153
Tb.Sp (mm)	0.60 ± 0.18	0.51 ± 0.13	0.021
Tb1.NSD (mm)	0.32 ± 0.21	0.29 ± 0.31	0.385

* Results expressed as the mean ± standard deviation (SD) values of the measured parameters. vBMD = volumetric bone mineral density; Dtrab = volumetric trabecular bone mineral density; Dinn = inner trabecular bone density; Dmeta = metatrabecular bone density; Dcort = volumetric cortical bone mineral density; D100 = total volumetric bone mineral density; Tb.Th = trabecular thickness; Tb.N = trabecular number; Tb.Sp = trabecular separation; Ct.Th = cortical thickness; Tb1.NSD = intra-distribution individual separation; BV/TV = trabecular bone volume.

Relationship between systemic sclerosis characteristics and bone parameters assessed by univariate regression analysis are presented in Table 5. In addition, among qualitative variables, presence of anticentromere antibodies (ACAs) was associated with BMD at all sites (*p* < 0.01), with Dtrab at radius and tibia (*p* < 0.05) and BV/TV (*p* < 0.05). History or current digital ulcers were associated with TH BMD (*p* < 0.05), Dtrab at tibia (*p* < 0.05) and with Tb.N at tibia (*p* < 0.05). Variables with *p* < 0.2 were selected for multivariate analyses.

The multivariate analyses showed that variables explaining independently Dtrab at the tibia (r^2^ = 0.33; *p* = 0.007) were ACAs, history of digital ulcers, lean mass and number of previous fracture. Variables explaining Tb.Sp at tibia (r^2^ = 0.66, *p* < 0.0001) were current digital ulcers, number of previous fracture, lean mass and menopause duration. Variables independently influencing TH BMD (r^2^ = 0.73; *p* < 0.00001) were age, lean mass and ACAs. The same variables also explained LS and FN BMD however with smaller r^2^ values in these models.

**Table 5:** Relationship between systemic sclerosis characteristics and bone parameters, by univariate regression analysis*

	Age	BMI	Menopause duration	Disease duration	Fat mass	Lean mass	Number of previous ostoporotic fractures
BMD (g/cm^2^)							
LS	−0.37*	0.3	NS	0.28	0.36	0.43*	NS
FN	−0.48*	0.60**^╪^**	NS	NS	0.54**^┼^**	0.72**^╪^**	NS
TH	−0.48*	0.57**^╪^**	−0.33	NS	0.51**^┼^**	0.77**^╪^**	−0.31
Dtrab (mg HA/cm^3^)							
Radius	−0.46*	0.34	−0.47*	NS	0.32	0.45*	−0.37*
Tibia	−0.34	0.28	−0.33	NS	NS	0.13*	−0.44*
Tb.N tibia (1/mm)	−0.39	0.33	−0.35	0.42*	0.38*	0.43*	−0.49**^┼^**
Tb.Th radius (mm)	NS	NS	NS	NS	NS	NS	NS
Tb.Sp (mm)							
Radius	0.43*	−0.41*	0.47*	0.32	−0.31	−0.47^┼^	0.47^┼^
Tibia	0.43*	−0.37*	0.45*	−0.31	−0.41*	−0.39*	0.56^┼^
BV/TV							
Radius	−0.46*	0.33	−0.47*	NS	0.32	0.45*	−0.37*
Tibia	−0.33	0.28	−0.33	NS	0.24	0.4	−0.44*

Results are given with the correlation coefficient r.

NS = not significant.

* p <0.05.

┼ p <0.01.

╪ p <0.001.

BMI = body mass index; BMD = bone mineral density; Dtrab = trabecular volumetric bone mineral density; Tb.N = trabecular number;

Tb.Th = trabecular thickness; Tb.Sp = trabecular separation; BV/TV = trabecular bone volume.

## DISCUSSION

The prevalence of OP was 3 to 51.1% in a systematic review of bone status in patients with SSc [19]. In the present study, the prevalence of OP was 42.8 % in postmenopausal women with SSc and was significantly higher than in controls. We have found significantly lower BMD values at all sites in patients compared to controls. In addition, osteopenia was more frequently observed at total hip in patients versus controls (Table 2). Our results are in line with previous studies [8, 20]. Note that the range of values of OP prevalence in SSc reported in the literature is rather large due to different population characteristics (age, menopausal status, organ involvement, gender, disease subtype and daily GC dose) [19, 20].

Previous studies in SSc patients have shown that low BMI was associated with low BMD [6, 8, 20, 21]. Body composition and more specifically lean mass has been shown to be a significant factor associated with FN and LS BMD [8] and it was demonstrated that BMI constitutes the main determinant influencing BMD [21]. Accounting for these data, we made a match on BMI. Despite this match analysis, BMI remained significantly lower in the patients group. After adjustment of densitometric values on this parameter, it remained a significant difference between the two groups for TH BMD. However, we did not detect any significant differences between the two groups in fat mass or lean mass.

To our knowledge, this is the first study that assessed the alteration of BMD and parameters of bone microarchitecture between SSc patients and healthy subjects.

This is the primary data that showed a significant alteration of trabecular bone compartment in patients with SSc. This preferential alteration of trabecular bone was also demonstrated in rheumatoid arthritis [22]. In contrast, in systemic lupus erythematosus patients treated with long-term GC, it has been found an unexpected deterioration of the microarchitecture which predominated at the cortical bone, with no significant difference at the trabecular compartment [23].

## MATERIALS AND METHODS

### Study population

A total of thirty-three SSc patients and 33 controls (details below) were recruited during a 10 months period (April 2012 to February 2013) from Rheumatology and Dermatology departments of the Centre Hospitalier Régional d'Orléans (France). All SSc patients fulfilled criteria of the American College of Rheumatology and/or criteria of LeRoy and Medsger [14]. Exclusion criteria were underage, pregnant or breastfeeding women and lack of understanding French language (for consent).

Likewise we included healthy women matched one to one with patients on age (+/− 4 years), body mass index (BMI) (+/− 3 kg/m²) and duration of menopause (+/− 1 year). Controls were selected from a database obtained from voluntary control women members of complementary health insurance (MGEN, MNH France). Exclusion criteria for controls were factors that may cause bone loss, such as use of glucocorticoids (GC) for a period at least 3 consecutive months with daily doses above 7.5 mg/day equivalent prednisone, an untreated hyperthyroidism, a primary hyperparathyroidism, a chronic disease such as renal failure or chronic respiratory failure, and a chronic inflammatory rheumatic disease. In addition, women who had used anti-osteoporotic treatments were excluded.

The study was approved by the local Ethical Committee (No. 2012- R1). Written informed consent was obtained from all subjects.

### Clinical evaluation

Information concerning age, weight, height, menopause duration, traditional OP risk factors and use of medications, were collected from patients and controls. In patients, disease characteristics were reported: disease duration, disease subtype (limited or diffuse cutaneous SSc, or sine scleroderma SSc), overall functional disability measured with the Health Assessment Questionnaire (HAQ) and current treatments (Table 1). Skin involvement was assessed by the modified Rodnan skin thickness score (mRSS) [15]. The vascular disease was defined by presence of Raynaud syndrome and history or current digital ulcers. Joint damage was defined by history or current arthralgia or arthritis. Lung disease was defined by presence of abnormalities on functional exploration, such as diffusing capacity for carbon monoxide lower than 75% or a total lung capacity lower than 80% and/or the presence of interstitial lung disease seen on chest X-Ray and/or by the presence of pulmonary hypertension. Gastrointestinal involvement was defined by presence of dysphagia, gastroesophageal reflux, malabsorption syndrome and damage to oesophageal manometry. Renal involvement was assessed by serum creatinine and history of scleroderma renal crisis.

### Biological evaluation

Fasting serum samples were collected from all patients and used to analyse laboratory parameters displayed in Table 1.

### Bone mineral density (BMD) assessment

BMD was assessed by DXA on a Discovery instrument (Hologic) at the lumbar spine (LS) (L1-L4, anterior projection), femoral neck (FN) of the nondominant side, total hip (TH) and whole body. Vertebral Fracture Assessment was performed if the patient complained of back pain and/or when there was either prospective height loss greater than 2 cm, or an historical height loss greater than 4 cm [16]. The Word Health Organization definition of osteoporosis was applied [17]. For premenopausal patients, bone density was expressed as Z-score. All measurements of BMD were performed by experienced technicians.

### High-Resolution peripheral Quantitative Computed Tomography (HR-pQCT) measurements

HR-pQCT (XtremCT; Scanco Medical AG, Brüttisellen, Switzerland) provided access to vBMD and bone microarchitecture *in vivo* at the nondominant tibia and radius. All patients and controls were imaged using the manufacturer standard *in vivo* protocol as described previously [10]. The maximum resolution achieved by this technique was 82 × 82 × 82 μm3. Images were processed using the default clinical evaluation protocol provided by the manufacturer to derive standard cortical and trabecular geometric and density measures [18]. During the examination, the arm and the leg of the subject were immobilized in a splint made of carbon fiber. An anteroposterior scout view was used to define the measured region. The effective dose was 3 μSv per measure in 3 minutes.

The entire volume of interest was automatically separated into a cortical and trabecular region using a threshold-based algorithm [18]. The outcome variables used in our analyses were the following : vBMD volumetric bone mineral density (mg HA/cm3), Dtrab volumetric trabecular bone mineral density (mg HA/cm3), Dinn inner trabecular bone density (mg HA/cm3), Dmeta metatrabecular bone density (mg HA/cm3), Dcort volumetric cortical bone mineral density (mg HA/cm3), D100 total volumetric bone mineral density (mg HA/cm3), Tb.Th trabecular thickness (mm), Tb.N trabecular number (mm-1); Tb.Sp trabecular separation (mm), Ct.Th cortical thickness (mm); Tb1.NSD intra-distribution individual separation (mm), BV / TV trabecular bone volume (%).

One patient was excluded owing to excessive motion artefact.

### Statistical analysis

Analyses were performed using the R software Version 2.15.2. For the comparative study of parameters measured by DXA and HR-pQCT, means were presented with their standard deviation (SD). Means measured in patients were compared to those observed in control, using the Student's paired samples t test (Wilcoxon rank-sum test for skewed distributed variables). *p* values < 0.05 were considered to be significant. For the univariate analysis of the relationship between bone parameters and disease characteristics, quantitative variables were studied by the Pearson correlation coefficient (Spearman's rank correlation coefficient for skewed distributed variables). Analysis of variance was used for qualitative variables. Variables with p< 0.2 were selected for multivariate analyses. A multivariate linear regression analysis was performed to model the relationship between some bone architecture variables and patients or disease characteristics. The main benchmark for choosing the best model was the adjusted coefficient R². Normality of residuals was verified by graphical method and controlled by the Shapiro-Wilk normality test. Equivariance and independence of residuals were tested by the Residues-Adjusted Values graph.

Several factors may contribute and influence the risk of OP in SSc. Systemic inflammation is a causal factor often mentioned to have some relevance in the induction of OP [5]. However although a high erythrocyte sedimentation rate (ESR) (>30mm/1^st^ h) is considered to be a relevant disease activity variable [24], an inflammatory syndrome with high C reactive Protein (CRP) levels is an unusual presentation in patients with SSc. Only 3 patients in our study exhibited an inflammatory syndrome (higher CRP and/or ESR), preventing us to analyse the relationship between BMD and systemic inflammation. Furthermore, gastrointestinal involvement is frequent in SSc and malabsorption may be also responsible for calcium and vitamin D deficiency. Regarding vitamin D, it has been demonstrated that deficiency and insufficiency were respectively present in 23 to 32% and 82 to 86% in Italian and French cohorts of patients with SSc [25]. These last results contrasted with our data showing less vitamin D deficiency and insufficiency (Table 1). In addition, we did find neither significant relationship between low values of vitamin D and BMD values nor with fracture events. This can be explained by the small number of patients with fractures (*n* = 10).

We highlighted in multivariate analyses, an association between ACAs and bone parameters on the one hand, and digital ulcers and bone changes on the other hand. Indeed, the presence of ACAs was significantly associated in our study with low BMD values at all sites, and lower values of Dtrab. This data has never been reported in the literature. Ibn Yacoub Y *et al*. [26] emphasized the link between the decrease in BMD and the presence of anti-ScL70, suggesting a relationship between BMD and the diffuse subset of SSc. This link between the diffuse subset and low BMD was also highlighted by other authors [6, 7, 19]. Herein, we did not found parallel relationship between this form and the deterioration of bone tissue. This can be explained by the small number of patients with diffuse cutaneous subset (*n* = 3).

Furthermore, in multivariate analyses, previous and current digital ulcers were associated respectively and significantly to a lower value of Dtrab and higher value of Tb.Sp at tibia. In univariate analysis, digital ulcers were also associated with lower FN and TH BMD values. It should be noted that among the ten patients who had one or more cyclic intravenous prostanoids treatments, eight had OP and/or osteopenia. This suggested that the presence of severe digital ulcers is associated with low BMD. It could be speculated that the vascular changes may play a role in the process of demineralization. The existence of digital ulcers may reflect systemic microangiopathy and may be involved in the alteration of bone tissue. The link between digital ulcers and acro-osteolysis has been show [4, 27]. Repeated vasospasm [4, 27] have been suspected to play a role in the bone resorption process. In addition, a link between decreased FN, ankle and foot BMD, and an ipsilateral lower limb arterial disease has been shown in the study of Laroche M *et al* [28]. The association between ACAs and digital ulcer has been shown in several studies [29, 30]. This could suggest that the direct or indirect pathogenic role of ACAs may occur on bone, via a vascular disease.

This study presents a novel unique approach as it examines both the bone status in SSc patients using HR-pQCT, but also the influence of demographic and clinical factors on BMD and microarchitectural parameters. Moreover, the matching control method provides the advantage of reducing confusing factors such as age and duration of menopause. Indeed, in previous studies, the risk factors associated with low BMD were older age [6, 19, 20, 31] and duration of menopause [8, 19, 20, 32]. Another strength is the fact that the study population is relatively homogeneous in terms of the subtype of the disease: 26/33 patients had limited cutaneous form. Thus, all results could be applicable to this category of patients. The main limitation of our study is the small size of our population (*n* = 33) as compared to a nationwide population-based study [33]. However, SSc is a rare disease and the number of included patients in our study is relative, compared to the number of previous studies concerned with bone damage in SSc. Inclusion of patients treated with GC, inhibitor proton pumps and other immunosuppressors may be a confounding factor, because these treatments are likely to act on bone metabolism. However, in univariate analysis, these treatments were not associated with any of the bone parameters. Moreover, GC did not appear in any of our models as an explanatory factor. There is a scientific debate about the impact of GC on BMD and OP in SSc: it has been showed that GC did not influence the occurrence of OP in SSc [34] and that cumulative GC doses did not differ between women with or without osteoporosis fractures, which is in contrast with the role of GC in the occurrence of fractures reported by Lai *et al.* [33].

In conclusion, our study showed an increased prevalence of OP in patients with SSc compared with a control group. Both bone density and microarchitecture alteration predominated at trabecular compartment. Moreover, this is the first study demonstrating on one hand, a significant relationship between digital ulcers and alterations of bone structure and on the other hand, the association between ACAs and alteration of bone tissue. Presence of ACAs, and history or current digital ulcers in post menopausal women with SSc, might lead to a densitometry test.

## References

[1] Gabrielli A, Avvedimento EV, Krieg T, Scleroderma N (2009). Engl J Med.

[2] Hudson M, Thombs BD, Steele R, Panopalis P, Newton E, Baron M (2009). Health-related quality of life in systemic sclerosis: a systematic review. Arthritis Rheum.

[3] Sandqvist G, Eklund M, Akesson A, Nordenskiöld U (2004). Daily activities and hand function in women with scleroderma. Scand J Rheumatol.

[4] Avouac J, Mogavero G, Guerini H, Drapé JL, Mathieu A, Kahan A, Allanore Y (2011). Predictive factors of hand radiographic lesions in systemic sclerosis: a prospective study. Ann Rheum Dis.

[5] Roux C (2011). Osteoporosis in inflammatory joint diseases. Osteoporos Int J.

[6] Di Munno O, Mazzantini M, Massei P, Ferdeghini M, Pitaro N, Latorraca A, Ferri C (1995). Reduced bone mass and normal calcium metabolism in systemic sclerosis with and without calcinosis. Clin Rheumatol.

[7] Frediani B, Baldi F, Falsetti P, Acciai C, Filippou G, Spreafico A, Siagri C, Chellini F, Capperucci C, Filipponi P, Galeazzi M, Marcolongo R (2004). Clinical determinants of bone mass and bone ultrasonometry in patients with systemic sclerosis. Clin Exp Rheumatol.

[8] Souza RBC, Borges CTL, Takayama L, Aldrighi JM, Pereira RM (2006). Systemic sclerosis and bone loss: the role of the disease and body composition. Scand J Rheumatol.

[9] K Kowal-Bielecka O1, Landewé R, Avouac J, Chwiesko S, Miniati I, Czirjak L, Clements P, Denton C, Farge D, Fligelstone K, Földvari I, Furst DE, Müller-Ladner U (2009). EULAR recommendations for the treatment of systemic sclerosis: a report from the EULAR Scleroderma Trials and Research group (EUSTAR). Ann Rheum Dis.

[10] Boutroy S, Bouxsein ML, Munoz F, Delmas PD (2005). *in vivo* assessment of trabecular bone microarchitecture by high-resolution peripheral quantitative computed tomography. J Clin Endocrinol Metab.

[11] Sornay-Rendu E, Boutroy S, Munoz F, Delmas PD (2007). Alterations of cortical and trabecular architecture are associated with fractures in postmenopausal women, partially independent of decreased BMD measured by DXA: the OFELY study. J Bone Miner Res.

[12] Vico L, Zouch M, Amirouche A, Frère D, Laroche N, Koller B, Laib A, Thomas T, Alexandre C (2008). High-resolution pQCT analysis at the distal radius and tibia discriminates patients with recent wrist and femoral neck fractures. J Bone Miner Res.

[13] Tang XL, Qin L, Kwok AW, Zhu TY, Kun EW, Hung VW, Griffith JF, Leung PC, Li EK, Tam LS (2013). Alterations of bone geometry, density, microarchitecture, and biomechanical properties in systemic lupus erythematosus on long-term glucocorticoid: a case-control study using HR-pQCT. Osteoporos Int J.

[14] LeRoy EC, Medsger TA (2001). Criteria for the classification of early systemic sclerosis. J Rheumatol.

[15] Lachenbruch PA, Seibold JR, Zee B, Steen VD, Brennan P, Silman AJ, Allegar N, Varga J, Massa M (1993). Skin thickness score in systemic sclerosis: an assessment of interobserver variability in 3 independent studies. J Rheumatol.

[16] Schousboe JT, Vokes T, Broy SB, Ferrar L, McKiernan F, Roux C, Binkley N (2008). Vertebral Fracture Assessment: the 2007 ISCD Official Positions. J Clin Densitom.

[17] Kanis JA, WHO Study Group Assessment of fracture risk and its application to screening for postmenopausal osteoporosis: synopsis of a WHO report. Osteoporos Int J.

[18] Laib A, Häuselmann HJ, Rüegsegger P (1998). *In vivo* high resolution 3D-QCT of the human forearm. Technol Health Care.

[19] Omair MA, Pagnoux C, McDonald-Blumer H, Johnson SR, A systematic review (2013). Low bone density in systemic sclerosis. J Rheumatol.

[20] Mok CC, Chan PT, Chan KL, Ma KM (2005). Prevalence and risk factors of low bone mineral density in Chinese patients with systemic sclerosis: a case-control study. Rheumatology (Oxford).

[21] Sampaio-Barros PD, Costa-Paiva L, Filardi S, Sachetto Z, Samara AM, Marques-Neto JF (2005). Prognostic factors of low bone mineral density in systemic sclerosis. Clin Exp Rheumatol.

[22] Fouque-Aubert A, Boutroy S, Marotte H, Vilayphiou N, Bacchetta J, Miossec P, Delmas PD, Chapurlat RD (2010). Assessment of hand bone loss in rheumatoid arthritis by high-resolution peripheral quantitative CT. Ann Rheum Dis.

[23] Tang XL, Qin L, Kwok AW, Zhu TY, Kun EW, Hung VW, Griffith JF, Leung PC, Li EK, Tam LS (2013). Alterations of bone geometry, density, microarchitecture, and biomechanical properties in systemic lupus erythematosus on long-term glucocorticoid: a case-control study using HR-pQCT. Osteoporos Int J.

[24] Valentini G, Della Rossa A, Bombardieri S, Bencivelli W, Silman AJ, D'Angelo S European multicentre study to define disease activity criteria for systemic sclerosis. II. Identification of disease activity variables and development of preliminary activity indexes. Ann Rheum Dis.

[25] Vacca A, Cormier C, Mathieu A, Kahan A, Allanore Y Vitamin D levels and potential impact in systemic sclerosis. Clin Exp Rheumatol.

[26] Ibn Yacoub Y, Amine B, Laatiris A, Wafki F, Znat F, Hajjaj-Hassouni N (2012). Bone density in Moroccan women with systemic scleroderma and its relationships with disease-related parameters and vitamin D status. Rheumatol Int.

[27] Avouac J, Guerini H, Wipff J, Assous N, Chevrot A, Kahan A, Allanore Y (2006). Radiological hand involvement in systemic sclerosis. Ann Rheum Dis.

[28] Laroche M, Moulinier L, Leger P, Lefebvre D, Mazières B, Boccalon H (2003). Bone mineral decrease in the leg with unilateral chronic occlusive arterial disease. Clin Exp Rheumatol.

[29] Chung L, Utz PJ (2004). Antibodies in scleroderma: direct pathogenicity and phenotypic associations. Curr Rheumatol Rep.

[30] Bolster L, Taylor-Gjevre RM, Nair B, Gjevre JA (2010). Digital gangrene associated with anticentromere antibodies: a case report. J Med Case Reports.

[31] Yuen SY, Rochwerg B, Ouimet J, Pope JE (2008). Patients with scleroderma may have increased risk of osteoporosis. A comparison to rheumatoid arthritis and noninflammatory musculoskeletal conditions. J Rheumatol.

[32] La Montagna G, Baruffo A, Abbadessa S, Maja L, Tirri R (1995). Evidence for bone resorption in systemic sclerosis. J Rheumatol.

[33] Lai C-C, Wang S-H, Chen WS, Liu CJ, Chen TJ, Lee PC, Chang YS (2014). Increased risk of osteoporotic fractures in patients with systemic sclerosis: a nationwide population-based study. Ann Rheum Dis.

[34] Avouac J, Koumakis E, Toth E, Meunier M, Maury E, Kahan A, Cormier C, Allanore Y (2012). Increased risk of osteoporosis and fracture in women with systemic sclerosis: a comparative study with rheumatoid arthritis. Arthritis Care Res.

